# Cost-Effectiveness of Blood-Based Brain Biomarkers for Screening Adults with Mild Traumatic Brain Injury in the French Health Care Setting

**DOI:** 10.1089/neu.2022.0270

**Published:** 2023-03-28

**Authors:** Louise Zimmer, Cheryl McDade, Hadi Beyhaghi, Molly Purser, Julien Textoris, Alexander Krause, Esther Blanc, Vladislav Pavlov, Stephanie Earnshaw

**Affiliations:** ^1^ bioMérieux, Inc., Durham, North Carolina, USA.; ^2^ RTI Health Solutions, Research Triangle Park, North Carolina, USA.; ^3^ bioMérieux, SA, Marcy-l'Étoile, France.

**Keywords:** biomarker, concussion, cost-effectiveness, costs, mild traumatic brain injury

## Abstract

Two blood-based brain biomarker tests such as the combination of glial fibrillary acidic protein and ubiquitin C-terminal hydrolase-L1 (GFAP+UCH-L1) or S100B have potential to reduce the need for head computed tomography (CT) scanning in patients with mild traumatic brain injury (mTBI). We assessed the clinical and economic impact of using GFAP+UCH-L1 versus CT scan and GFAP+UCH-L1 versus S100B to screen adults with suspected mTBI presenting to an emergency department (ED). A decision model was developed to estimate costs and health outcomes of GFAP+UCH-L1, CT scan, and S100B associated with these screening protocols. Model parameters were extracted from peer-reviewed articles, clinical guidelines, and expert opinion. Analysis was performed from a French health care system perspective (costs in 2020 euros). In the model, patients with a positive biomarker receive a CT scan to confirm the presence of intracranial lesions (ICLs). Depending on clinical state and biomarker and CT results, patients were discharged immediately, kept for observation in the ED, admitted for in-hospital stay and observation, or admitted for surgical management. Incorrect test results may lead to delayed treatment and poor outcomes or overtreatment. GFAP+UCH-L1 use was associated with an overall decrease in CT scans when compared with CT screening or S100B use (325.42 and 46.43 CTs per 1000 patients, respectively). The use of GFAP+UCH-L1 resulted in modest cost savings when compared with CT scanning and with S100B. In all cases, use of GFAP+UCH-L1 marginally improved quality-adjusted life-years (QALYs) and outcomes. Thus, screening with GFAP+UCH-L1 reduced the need for CT scans when compared with systematic CT scan screening or use of S100B while maintaining similar costs and health outcomes.

## Introduction

According to the World Health Organization, mild traumatic brain injury (mTBI) is a head injury defined by a Glasgow Coma Scale (GCS) score of 13–15 at 30 min post-injury and one or more of the following symptoms: loss of consciousness for <30 min, post-traumatic amnesia presentation for <24 h, impaired mental state (such as confusion or disorientation), and/or transient neurological deficit.^1,2^ Mild TBI is estimated to account for 80–90% of all cases of TBI in both civilian and military populations.^3^

Health care resource use is substantial in patients with mTBI. This may include but is not limited to emergency department (ED) visits, inpatient and outpatient observation and treatment, follow-up visits, and rehabilitation. In the acute post-traumatic period, almost half of all patients seeking care receive brain imaging, with 98% of the imaging being computed tomography (CT) scans, a reference method to detect acute post-traumatic intracranial lesions (ICLs).^4^

Although head CT scans can detect acute post-traumatic ICLs and thus identify patients at risk for further deterioration that may eventually require medical or surgical treatment, most patients for whom CT scan is used to evaluate mTBI do not have detectable ICLs. A systematic review of studies including more than 23,000 patients with a GCS score of 13–15 found that the prevalence of severe intracranial injury that required prompt intervention was 7.1% (95% confidence interval, 6.8%-7.4%), and the prevalence of injuries leading to death or requiring neurosurgical intervention was 0.9%.^5^ As such, CT scanning is overused and unnecessarily exposes many patients to radiation.

There is a growing body of evidence indicating that ICLs can be predicted by measuring brain-specific proteins released to human serum after a trauma-induced injury of brain cells.^6^ The availability of blood tests for mTBI could help clinicians determine the need for a CT scan and potentially prevent unnecessary neuroimaging and associated radiation exposure while bringing more objectivity to patient care. The blood-based biomarker S100B is Conformité Européenne (CE) marked; is used in clinical practice in several European countries, including France; and has been incorporated into the Scandinavian guidelines.^7,8^ Subsequently, a prospective study found that incorporating S100B into the guidelines for management of mTBI was cost saving.^8^

In 2018, combination of glial fibrillary acidic protein and ubiquitin C-terminal hydrolase-L1 (GFAP+UCH-L1, Banyan Biomarkers) was approved by the U.S. Food and Drug Administration (FDA),^9^ with subsequent publication of health economic data for US health care settings.^10^ Other manufacturers are developing a blood test and/or have received an FDA clearance of a blood test combining GFAP and UCH-L1^11^ followed by CE marking in Europe in 2021.^12^ However, health economic data on GFAP+UCH-L1 in Europe are lacking.

In this analysis, we evaluated the clinical and economic impact of using GFAP+UCH-L1 to screen adults presenting to an ED with suspected mTBI in France. Specifically, we examined the potential impact of the test if it was introduced to actual clinical practice and compared it with screening all patients with head CT or screening with the S100B biomarker.

## Methods

A decision-analytic model was developed in Microsoft Excel to examine costs and health outcomes associated with the use of blood-based brain biomarker tests and CT scanning for screening patients presenting to the ED with suspected mTBI. The model was developed from a French health care perspective with a lifetime time horizon. Costs are presented in 2020 euros. Costs and outcomes were discounted at 2.5% up to 30 years and 1.5% thereafter.^13^ Details are available in Supplementary Appendix S1.

All data used to populate the model are publicly available, and no patient-level data were used in this model. Thus, review/approval by an institutional review board was not required.

### Patient population

The target patient population was adults age ≥18 years who presented to the ED with suspected mTBI (GCS score of 13–15) within 12 h of injury.^7,14^

### Comparators

Biomarkers in this analysis included S100B or GFAP+UCH-L1 along with standard clinical assessment for mTBI. GFAP and UCH-L1 are two different brain-specific protein biomarkers approved by the FDA for use within 12 h of injury in patients age ≥18 years with suspected mTBI.^15^ S100B has high clinical sensitivity for abnormal head CT scans in patients with isolated head trauma when measured within 6 h of head injury.^7,16^

In real-world practice, patients exhibiting symptoms of mTBI may arrive at the ED within 6 h of injury or later. Thus, the proportion of patients arriving during the first hours following injury will vary in different hospital settings. When using biomarkers with time window restrictions, hospitals need to revert to using CT scanning as the primary evaluation method when patients arrive outside the approved time window. As such, we evaluated a number of hospital scenarios in which all patients with mTBI arrive at the ED within 12 h of injury and are eligible for screening with one of the biomarkers, CT, or a combination of one of the biomarkers and CT.

**FIG. 1.**
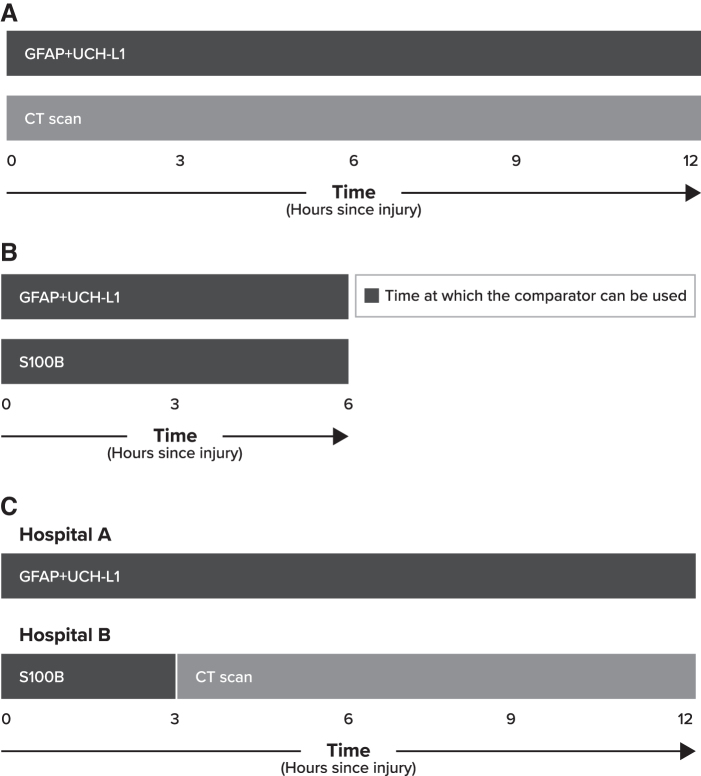
Screening comparisons. **(A)** GFAP+UCH-L1 versus CT scan. **(B)** GFAP+UCH-L1 versus S100B. **(C)** Hospital A with GFAP+UCH-L1 versus Hospital B with S100B and CT Scan. CT, computed tomography; GFAP+UCH-L1, glial fibrillary acidic protein and ubiquitin C-terminal hydrolase-L1.

Three comparisons were performed (Fig. 1):

- GFAP+UCH-L1 versus CT scan (Fig. 1A): combination GFAP+UCH-L1 versus all patients who undergo CT scanning within 12 h of injury
- GFAP+UCH-L1 versus S100B (Fig. 1B): combination GFAP+UCH-L1 versus S100B within 6 h of injury per S100B package insert^16^
- GFAP+UCH-L1 versus a combination of S100B and CT scan (Fig. 1C): a population-based analysis in which hospital A has access to the GFAP+UCH-L1 biomarker and hospital B has access to the S100B biomarker in which S100B is used within 3 h so that the number of false-negatives is minimized.^17^ Specifically, GFAP+UCH-L1 is used in hospital A for screening within 12 h following injury compared with hospital B in which 70%, 50%, or 30% of patients receive S100B within the first 3 h of injury and the remaining 30%, 50%, or 70% of patients receive a CT scan between 3 and 12 h following injury. Jehlé and colleagues^17^ reported in an expert consensus that even though approved for use within 6 h of injury, an S100B test performed within 3 h allows for maximal sensitivity (close to 100%).

### Model structure

**FIG. 2.**
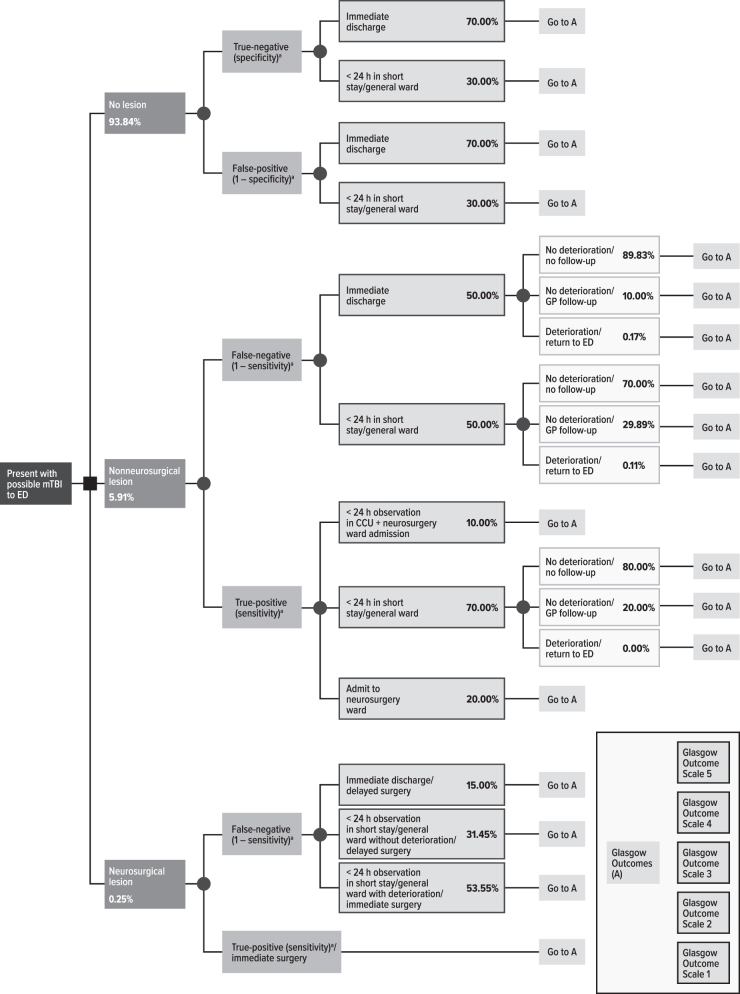
Model structure. ^a^Value in parentheses represents probability of proceeding through this branch and varies by model comparator. CCU, critical care unit; ED, emergency department; GP, general practitioner; mTBI , mild traumatic brain injury.

The model is a decision tree structure (Fig. 2) to simulate short- and long-term costs and outcomes. Patients enter the model by presenting to the ED with a suspected mTBI. At this visit, after clinical examination, patients are screened with a head CT or one of the biomarker tests to evaluate the presence or absence of lesions (i.e., non-neurosurgical lesions or lesions requiring surgical evacuation). Diagnostic performance of the CT scan and biomarker tests (i.e., sensitivity and specificity) is used to evaluate this presence. In patients who have a positive biomarker test result, a CT scan is performed to confirm the presence of ICLs. Depending on the biomarker test outcome and/or the CT results, patients may be discharged immediately from the ED, kept for <24-h observation, or admitted to a short-stay general ward or neurosurgery ward. Patients without lesions may be discharged immediately or kept for short-term observation.

Patients with lesions receiving a correct diagnosis (true-positive) are assumed to receive appropriate care and have optimal outcomes. Patients with lesions who are misdiagnosed (false-negative) may be treated properly based on clinical judgment. However, a small portion of these patients may be discharged, further deteriorate, and require readmission for further evaluation. For these patients, delays in appropriate treatment may lead to suboptimal health outcomes and increased costs. Patients requiring neurosurgical intervention may receive additional (follow-up) CT scans. Patients with false-positive biomarker test results are likely to get a head CT and thus incur additional costs. Patient flow within the decision tree is presented in Figure 2 and is based on clinical opinion of French health professionals (ED physicians, neurosurgeons, and intensivists) involved in the care of mTBI patients.

Lesion presence and appropriate and timely patient management will affect patient outcomes as measured by the Glasgow Outcome Scale (GOS). Post-discharge, patients are assumed to remain in their resulting GOS health state for the remainder of their lifetime. Costs and outcomes are accrued specific to these GOS health states and the resulting resource use incurred in the initial admission and/or readmission.

### Lesion prevalence

The prevalence of ICL types in patients with mTBI was obtained from the ALERT-TBI pivotal trial (Table 1).^14^ This prevalence was found to be similar to the prevalence reported by Smits and associates.^18^

**Table 1.** Prevalence, Sensitivity, and Specificity

Model parameter					Base-case estimate (range)		
Prevalence in GCS score 13–15	Base-case values^14^				Alternative values^18^		
No lesion	93.84% (97.06%-93.84%)				92.36% (84.64%-100.00%)		
Non-neurosurgical lesion	5.91% (2.81%-5.91%)				7.10% (6.26%-8.05%)		
Neurosurgical lesion	0.26% (0.12%-0.26%)				0.53% (0.33%-0.85%)		

Biomarker sensitivity/specificity	GFAP+UCH-L1 (GCS score 13-15)^19^	GFAP+UCH-L1 (GCS score 13-15) alternative values^20^	GFAP+UCH-L1 (GCS score 14-15)^14^	GFAP+UCH-L1 (GCS score 15)^20^	GFAP+UCH-L1 (real world)^21^	S100B^22^	S100B alternative values^16^
Specificity (no lesion)	0.365 (0.342-0.387)	0.404 (0382-0.427)	0.367 (0.345-0.390)	0.411 (0.387-0.434)	0.250 (0.200-0.300)	0.310 (0.270-0.360)	0.300 (0.290-0.310)
Sensitivity (non-neurosurgical lesion)	0.975 (0.929-0.995)	0.958 (0.906-0.982)	0.973 (0.924-0.994)	0.957 (0.896-0.983)	1.000 (0.820-1.000)	0.960 (0.920-0.980)	0.990 (0.960-1.000)
Sensitivity (neurosurgical lesion)	1.000 (0.800-1.000)	1.000 (0.800-1.000)	1.000 (0.631-1.000)	1.000 (0.800-1.000)	1.000 (0.820-1.000)	1.000 (0.800-1.000)	1.000 (0.800-1.000)

GCS, Glasgow Coma Scale; GFAP+UCH-L1, glial fibrillary acidic protein and ubiquitin C-terminal hydrolase-L1.

### Sensitivity and specificity

Sensitivity and specificity for GFAP+UCH-L1 was obtained from the prospective clinical study ALERT-TBI upon which the U.S. FDA's approval decision was based.^19^ Analyses using another diagnostic platform to measure GFAP+UCH-L1 in slightly reduced ALERT-TBI populations resulted in alternative sets of sensitivity and specificity values that were examined in sensitivity analysis.^14,20^ In addition, sensitivity and specificity values for the GFAP+UCH-L1 combination, using alternative GFAP and UCH-L1 cutoff values in a cohort of 349 adult patients with mTBI in a Level 1 U.S. trauma center, were examined for completeness.^21^

The sensitivity and specificity of S100B was obtained from a meta-analysis performed to examine the efficacy of various blood-based protein biomarkers for diagnosing TBI.^22^ The data from that study are similar to the sensitivity and specificity of S100B reported in the S100B product characteristics,^16^ which are examined in a sensitivity analysis. Sensitivity and specificity parameters are presented in Table 1.

As the reference method, we made the simplifying assumption that CT scans are 100% sensitive and specific.

### Patient outcomes

To estimate the percentage of patients within each GOS health state based on lesion type and deterioration, data were extracted from published literature. A summary of the GOS by branch of the decision tree is presented in Table 2.

**Table 2.** Percentage of Patients Expected to Be in Health States Defined by GOS Score

GOS outcome	No lesion (assumption)	Non-neurosurgical lesions without deterioration (assumption)	Non-neurosurgical lesion with deterioration^38,39^	Neurosurgical lesion immediate surgery^38,40,41^	Neurosurgical lesion delayed surgery^38,40,41^
GOS 5	100.00% (80.00%-100.00%)	100.00% (80.00%-100.00%)	84.84% (51.58%-100.00%)	80.88% (49.18%-100.00%)	74.05% (45.02%-100.00%)
GOS 4	0.00% (0.00%-5.00%)	0.00% (0.00%-5.00%)	8.84% (5.37 %-12.30%)	13.24% (8.05%-18.42%)	12.12% (7.37%-16.87%)
GOS 3	0.00% (0.00%-5.00%)	0.00% (0.00%-5.00%)	5.06% (3.08%-7.05%)	4.41% (2.68%-6.14%)	10.38% (6.31%-14.45%)
GOS 2	0.00% (0.00%-5.00%)	0.00% (0.00%-5.00%)	0.63% (0.38%-0.88%)	0.00% (0.00%-0.00%)	0.00% (0.00%-0.00%)
GOS 1	0.00% (0.00%-5.00%)	0.00% (0.00%-5.00%)	0.63% (0.38%-0.88%)	1.47% (0.89%-2.05%)	3.46% (2.10%-4.82%)

Post year 5	Percentage of patients with changed GOS health state at year 6 and beyond				
Year 1 health state	GOS 3		GOS 4		GOS 5
GOS 3	57.69%		28.85%		13.46%
GOS 4	9.23%		58.46%		32.31%
GOS 5	5.88%		19.61%		74.51%

GOS, Glasgow Outcome Scale.

In the base-case analysis, a patient's resulting GOS health state was assumed to remain constant for the remainder of their lifetime. However, consistent with an analysis by Pandor and colleagues,^23^ we allowed patients to move between health states after year 6 in sensitivity analysis. The percentage of patients moving from their resultant health state after the index event to a GOS 3, 4, or 5 health state is presented in Table 2. Owing to the absence of data on long-term follow-up of patients with a GOS 2, we conservatively assumed these individuals would stay in GOS 2 unless they died because of all-cause mortality.

### Radiation-induced cancer

After a scan, patients were assumed to have an increased lifetime risk of radiation-induced cancer. Stein and associates^24^ reported an increased lifetime risk of cancer in patients after exposure. We fit an exponential distribution to project risk by age and assumed that the risk increased after every additional scan. As some of the risk data were measured in children and brain biomarkers are approved for use only in adult patients with mTBI, sensitivity analysis was performed in which lifetime risk was projected based on only the last 2 risk data points among individuals who received CT scans at ages 15 and 20 years.

### Resource use and costs

Patients progressed through the model using various resources. Costs are reported in 2020 euros. Details are presented in Figure 2, Table 3, and Supplementary Appendix S1.

**Table 3.** Costs and Utilities

Model parameter		Base-case estimate^a^	Source/Assumption		
S100B		€32.40	Ministère des Solidarités et de la Santé^42^		
GFAP+UCH-L1		€32.40	Assumed parity with S100B due to absence of cost for GFAP+UCH-L1		
ED visit		€25.42	Agence technique de l'information sur l'hospitalisation^43^		
CT scan		€102.61	Includes cost of scan and radiologist review. Securite Sociale l'Assurance Maladie^44^		
Hospitalizations					
Continuous care unit (per day)		€323.27	Agence technique de l'information sur l'hospitalisation^43^		
Short stay for observation (<24 h)		€326.00	Agence technique de l'information sur l'hospitalisation^43^		
Neurosurgery ward (per day)		€1455.28	OECD,^45^ Picot, et al.^46^		
Physician resources					
ED physician		€30.04	Securite Sociale l'Assurance Maladie^47^		
General practitioner		€25.00	Securite Sociale l'Assurance Maladie^47^		
Neuroradiologist		€30.78	Securite Sociale l'Assurance Maladie^47^		
Neurologist		€39.00	Securite Sociale l'Assurance Maladie^47^		
Neuropsychologist		€50.00	Assumed same as neurosurgeon consultation due to availability of data		
Neurosurgeon		€50.00	Securite Sociale l'Assurance Maladie^47^		
Surgeries					
Burr hole		€6142.83	Agence technique de l'information sur l'hospitalisation^43^		
Craniotomy		€9312.77	Estimated as weighted average cost by trauma level (e.g., levels 1-4) and number of stays. Agence technique de l'information sur l'hospitalisation^43^		
Decompressive craniotomy		€13,365.67	Cost of craniotomy + cost of cranioplasty. Agence technique de l'information sur l'hospitalisation^43^ and Securite Sociale l'Assurance Maladie^48^		
GOS health state costs					
GOS 5 (good recovery)		€0.00	Assumption		
GOS 4 (moderate disability)		€21,169.91	INSEE,^49^ OECD,^45^ Pandor, et al.^23^		
GOS 3 (severe disability)		€41,821.68	INSEE,^49^ OECD,^45^ Pandor, et al.^23^		
GOS 2 (vegetative state - year 1)		€89,720.19	INSEE,^49^ OECD,^45^ Pandor, et al.^23^		
GOS 2 (vegetative state - years ≥2)		€57,483.79	INSEE,^49^ OECD,^45^ Pandor, et al.^23^		
GOS 1 (dead)		€0.00	Assumption		
Annual per-patient cancer cost		€10,059.33	COS Paris Healthcare,^50^ INSEE^49^		
Utility decrements					
ED/Hospital visit		0.012 (95% CI, 0.0050-0.0222)	Salomon, et al.^51^		
Cancer due to radiation exposure		0.103	Mittmann, et al.^52^		

Utility by GOS (95% CI)	Smits, et al.^2^	Aoki, et al.^53^	Dijkers^54^ QWB	Dijkers,^54^ Salomon, et al.^51^ HUI	Kosty, et al.^55^
GOS 5	0.88 (95% CI, 0.74-0.97)	0.85 (95% CI, 0.82-0.88)	0.80 (SE = 0.160)	0.93 (SE = 0.186)	0.93 (95% CI, 0.91-0.95)
GOS 4	0.51 (95% CI, 0.39-0.63)	0.63 (95% CI, 0.58-0.68)	0.53 (SE = 0.106)	0.48 (SE = 0.096)	0.75 (95% CI, 0.73-0.78)
GOS 3	0.15 (95% CI, 0.06-0.28)	0.26 (95% CI, 0.22-0.30)	0.43 (SE = 0.086)	0.13 (SE = 0.026)	0.50 (95% CI, 0.46-0.53)
GOS 2	0.00	0.08 (95% CI, 0.05-0.11)	0.00	0.00	0.11 (95% CI, 0.07-0.15)
GOS 1	0.00	0.00	0.00	0.00	0.00

^a^ Varied ±20% in sensitivity analyses.

CI, confidence interval; CT, computed tomography; ED, emergency department; GFAP+UCH-L1, glial fibrillary acidic protein and ubiquitin C-terminal hydrolase-L1; GOS, Glasgow Outcome Scale; HUI, health utilities index; QWB, quality of well-being; SE, standard error.

### Utilities

Utilities of GOS health states were obtained from the CT in Head Injury Patients (CHIP) study.^2^ Utility decrements were also considered for time spent in the ED/hospital and for occurrence of radiation-induced cancer (Table 3).

### Mortality

All-cause mortality was considered within the model by using age-specific, all-cause mortality obtained from the French National Vital Statistics.^25^ As there is a slight risk of death upon occurrence of the mTBI, death is considered an outcome of the index event and is captured through the GOS outcomes (Table 2).

Patients who experienced cancer due to exposure to radiation from the CT were assumed to have a slightly higher probability of death. In the same study in which Stein and associates^24^ examined the risk of radiation-induced cancer, the authors also reported a lifetime risk of mortality due to the occurrence of radiation cancer. An exponential distribution was fit to predict age-specific increased risk of mortality. All-cause mortality was then adjusted in these patients by increasing their risk. As with the radiation-induced cancer incidence, sensitivity analysis was performed in which risk was projected based on only the last 2 risk data points in individuals at ages 15 and 20 years.

### Model analysis

For each test, lifetime costs and outcomes were derived. One-way sensitivity, scenario, and probabilistic sensitivity analyses were run. Details are in Supplementary Appendix S1.

## Results

### Base-case results

When use of GFAP+UCH-L1 in all patients was compared with CT in all patients, those who were tested with GFAP+UCH-L1 received fewer CT scans (770.88 vs. 1096.30 per 1000 patients). The number of ED visits, years lived with favorable outcomes (GOS >3), life-years, and quality-adjusted life-years (QALYs) between the two tests were similar (Table 4, Supplementary Table S1), whereas the costs for GFAP+UCH-L1 were lower than costs for CT scanning by €4150 per 1000 patients. As a result, evaluation with GFAP+UCH-L1 is cost saving compared with CT scan.

**Table 4.** Costs and Outcome Results of GFAP+UCH-L1, CT Scan, and S100B

	GFAP+UCH-L1, patient arrival within 6 or 12 h	CT scan, patient arrival within 12 h	S100B, patient arrival within 6 h
Costs			
Biomarker	€32.40	€0.00	€32.40
Other diagnostic testing	€531.88	€568.43	€536.61
Total	€564.28	€568.43	€569.01
Outcomes per 1000 patients			
Number of initial scans	676.79	1000.00	724.55
Number of follow-up scans	94.09	96.30	92.76
Total number of scans	770.88	1096.30	817.32
Number of ED visits	1000.00	1000.00	1000.00
Years with favorable outcome^a^	35,284.71	35,284.72	35,284.71
Life-years	35,290.24	35,290.24	35,290.24
QALYs	30,697.76	30,697.73	30,697.75

^a^ Defined as years with GOS score >3.

Costs and outcome results shown are for when patients arrive at the ED within the respective time windows for each test.

CT, computed tomography; ED, emergency department; GFAP+UCH-L1, glial fibrillary acidic protein and ubiquitin C-terminal hydrolase-L1; GOS, Glasgow Outcome Scale; QALY, quality-adjusted life-year.

Assuming all patients arrived at the ED within 6 h such that all patients were eligible to be evaluated with GFAP+UCH-L1 or the S100B test, use of GFAP+UCH-L1 (vs. S100B) resulted in patients receiving fewer CT scans (770.88 vs. 817.32 per 1000 patients). The number of ED visits, years lived with favorable outcomes (GOS >3), life-years, and QALYs between the two tests were similar (Table 4, Table S1). For every 1000 patients, use of GFAP+UCH-L1 was associated with a €4736 cost reduction. As a result, evaluation with GFAP+UCH-L1 is considered cost saving (less costly and marginally more effective) compared with S100B.

### Scenario analysis results

In the hospital scenarios, hospital A has access to GFAP+UCH-L1, and as such, all patients arriving within 12 h of injury are tested with GFAP+UCH-L1 (Fig.1C, Table 5). If hospital B has S100B and 30% of patients arrive within 3 h of injury, hospital A required 242 fewer CT scans per 1000 patients. If more than 30% of patients arrive to the ED within 3 h of injury, the percentage of patients able to receive S100B increases and the need for CT scan decreases. Regardless of the percentage of patients arriving within 3 h of injury to hospital B (30%, 50% or 70%), the need for CT scans and total costs are lower in hospital A, which has access to GFAP+UCH-L1. In all scenarios, patients within both hospital A and hospital B experience similar favorable outcome (GOS >3), life-years, and QALYs.

**Table 5.** Costs and Outcome Results for Hospital A and Hospital B

	Hospital A All GFAP+UCH-L1	Hospital B		
		30% S100B/70% CT scan^a^	50% S100B/50% CT scan^a^	70% S100B/30% CT scan^a^
Costs				
Biomarker	€32.40	€9.72	€16.20	€22.68
Other	€531.88	€558.88	€552.52	€546.15
Total	€564.28	€568.60	€569.72	€568.84
Outcomes per 1000 patients				
Number of initial scans	676.79	917.37	862.28	807.19
Number of subsequent scans	94.09	95.24	94.53	93.83
Total number of scans	770.88	1012.61	956.81	901.01
Number of ED visits	1000.00	1000.00	1000.00	1000.00
Years with favorable outcome^b^	35,284.71	35,284.71	35,284.71	35,284.71
Life-years	35,290.24	35,290.24	35,290.24	35,290.24
QALYs	30,697.76	30,697.742	30,697.74	30,697.74

^a^ Percentage of patients tested with S100B or CT scan.

^b^ Defined as years with GOS score >3.

Hospital A: all patients receive GFAP+UCH-L1.

Hospital B: various proportions of patients arrive at the ED within 3 h of injury and receive S100B or arrive later and receive CT scan.

CT, computed tomography; ED, emergency department; GFAP+UCH-L1, glial fibrillary acidic protein and ubiquitin C-terminal hydrolase-L1; GOS, Glasgow Outcome Scale; QALY, quality-adjusted life-year.

Because GFAP+UCH-L1 is not yet marketed in France to the best of our knowledge, the actual cost of this test is not known. In a breakeven analysis, we estimated that GFAP+UCH-L1 could cost up to €36.55 and remain cost-neutral while incurring similar outcomes (favorable outcomes, life-years, and QALYs) when compared with CT scanning. When compared with S100B, GFAP+UCH-L1 could cost up to €37.14 and remain cost-neutral while incurring similar outcomes. Alternatively, assuming a cost of €32.40 for GFAP+UCH-L1, the cost of a CT scan could decline from €102.61 to €89.90 and evaluation with GFAP+UCH-L1 could be cost-neutral.

### Sensitivity analysis results

**FIG. 3.**
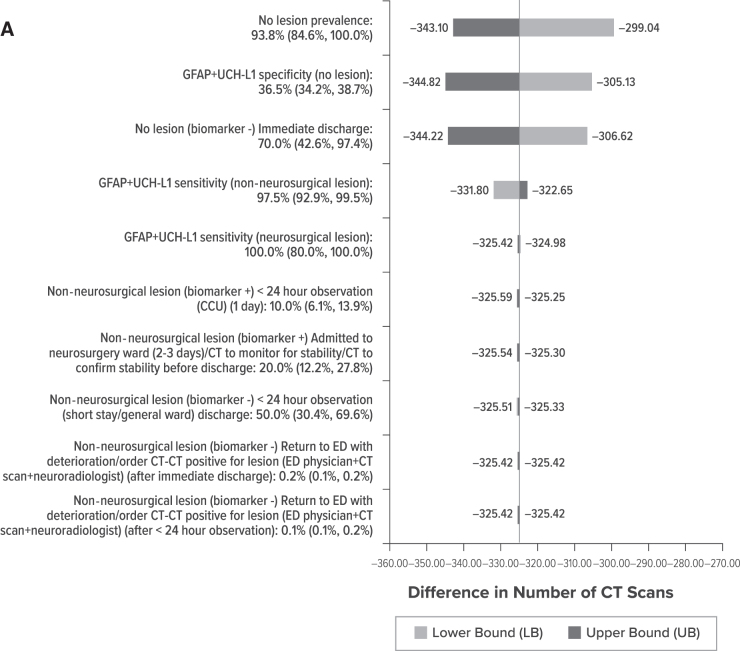

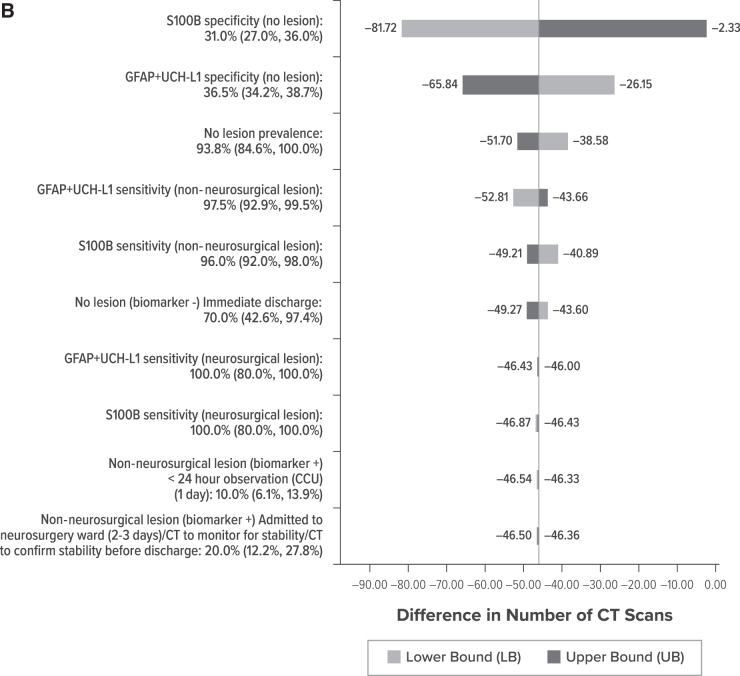
Impact of changes of each parameter on the difference in CT scans. **(A)** GFAP+UCH-L1 versus CT scan. **(B)** GFAP+UCH-L1 versus S100B. CCU, critical care unit; CT, computed tomography; ED, emergency department; GFAP+UCH-L1, glial fibrillary acidic protein and ubiquitin C-terminal hydrolase-L1.

When use of GFAP+UCH-L1 was compared with CT scan, one-way sensitivity analysis showed the difference in CT scans to be most sensitive to no lesion prevalence, GFAP+UCH-L1 specificity, and the proportion of patients being discharged immediately when no lesions exist (Fig. 3A). Use of GFAP+UCH-L1 reduced the need for CT scans within all plausible values.

When use of GFAP+UCH-L1 was compared with S100B, one-way sensitivity analysis showed the difference in CT scans to be most sensitive to GFAP+UCH-L1 and S100B specificity. Even with S100B's specificity at its upper bound or GFAP+UCH-L1's specificity at its lower bound, the number of CT scans incurred by patients using GFAP+UCH-L1 remained lower than the number of CT scans incurred by patients using S100B (Fig. 3B).

The use of the alternative sets of sensitivity and specificity values for GFAP+UCH-L1 (GCS 13–15) reduced the number of CT scans (from 771 in the base case to 734) and costs (from €564.28 per person in the base case to €559.78).

Because a more selective use of biomarkers cannot be excluded,^14^ we performed analysis of CT use and costs in GCS 14–15 and GCS 15 populations. When considering sensitivity and specificity values for GFAP+UCH-L1 in a GCS 14–15 population, both the number of CT scans (from 771 in the base case to 769) and costs (from €564.28 per person in the base case to €564.00) were reduced slightly. When considering sensitivity and specificity values for GFAP+UCH-L1 in a GCS 15 population, fewer CT scans (1096 when using CT scan to 728 when using GFAP+UCH-L1) and lower costs (from €568.43 per person when using CT scan and €559.06 per person when using GFAP+UCH-L1) remained. Thus, both costs and outcomes were improved with GFAP+UCH-L1 when compared with CT scan and S100B.

When considering sensitivity and specificity values for GFAP+UCH-L1 as extracted from a prospective cohort study from a Level 1 trauma center,^21^ fewer CT scans (1096 when using CT scan to 876 when using GFAP+UCH-L1) remained. However, the per-person cost increased (from €568.43 per person when using CT scan to €576.50 per person when using GFAP+UCH-L1).

The use of an alternative set of sensitivity and specificity values for S100B (obtained from the S100B product label) increased the difference in the number of CT scans (830 for S100B vs. 771 for GFAP+UCH-L1) and the difference in costs (€6,943 per 1,000 patients). Other outcomes, including ED visits, percentage with favorable outcome, life-years, and QALYs, remained unchanged.

Additional findings are presented in Supplementary Appendix S1.

## Discussion

The GFAP+UCH-L1 test is a novel test for ruling out the presence of ICLs on head CT scan in patients with mTBI. Use of this test as an aid in mTBI evaluation in our model was shown to decrease both the need for CT scans and costs while outcomes such as percentage with favorable outcome, life-years, and QALYs did not differ, which was expected given the limited knowledge about costs and outcomes associated with managing mTBI. Given the wide variation in definitions of mTBI in epidemiological studies, estimates of TBI incidence vary greatly.^1^ However, there is general agreement that in high-income countries rates are increasing among pediatric and elderly populations. In 2016, the incidence of mTBI in France, obtained from a nationwide hospital database, was 175 per 100,000 hospitalized patients.^26^ Extrapolating from an ED surveillance system to the entire nation, the 5-year average estimated incidence is 243.5 per 100,000 population.^27^ Thus, with the increasing incidence of mTBI, even small decreases in costs and improved outcomes can have a significant impact on a health care system.

The results of this analysis showed a decrease in CT scans when screening with GFAP+UCH-L1. This reduction may represent a significant benefit for hospitals and the health care system, as well as for patients, by reducing exposure to unnecessary radiation. Because CT scanning may require extra personnel for patient transfer and manipulation/movement of patients, GFAP+UCH-L1 could be useful in situations where these resources are unavailable or limited. This decrease in CT scan use may also free up this resource for patients with other pathologies (i.e., those more in need of emergent imaging such as stroke patients). In addition, it could decrease ED waiting times for patients who may not really require a CT scan. Specifically, evaluation and treatment may be based on clinical criteria and biomarker results, and thus, patients could avoid transport to and set up of the scan, technician time to perform the scan, as well as expert reading and interpretation by a radiologist. Further, as extended ED waiting times have been associated with lower patient satisfaction, such reductions could improve patient satisfaction and possibly reimbursement rates that may be linked to this satisfaction.^28^ However, to accomplish these benefits in the ED setting, obtaining results from these tests in a timely fashion is critical.

A number of economic analyses of novel biomarkers for detecting mTBI have been performed.^10,23,29^ Ruan and co-workers^29^ and Pandor and colleagues^23^ assessed S100B with standard care in the United States and the United Kingdom, respectively. Su and associates^10^ assessed the use of a GFAP+UCH-L1 test in comparison with usual care in U.S. patients with mild and moderate TBI. No economic analysis exists that compares the S100B, the GFAP+UCH-L1 combination (with different cutoffs and performance in sensitivity analysis), and CT scan approach all in one study.

Our analysis is structured similarly to that by Pandor and colleagues^23^ in that we examined costs and outcomes while considering various lesion types (no lesions, non-neurosurgical lesions, and neurosurgical lesions) along with the impact on longer-term outcomes/functional status. Results between these analyses are similar, with small differences in costs and QALYs. Absolute QALYs in our analysis align with those seen in the assessment by Ruan and co-workers.^29^ However, corresponding values were higher in our analysis than those seen in the study by Pandor and colleagues^23^ because of the distribution among GOS health states for patients with non-neurosurgical lesions with deterioration and those with neurosurgical lesions and movement between health states post-hospitalization.^23^ The patients in our analysis were more heavily distributed to higher GOS health states (better outcomes) because we adjusted for initial GCS score (i.e., GCS score of 13–15). Pandor and colleagues^23^ derived these distributions using clinical studies that included patients with more severe TBI (GCS <13), which showed use of biomarkers to be more economically favorable. In the study by Su and associates, only patients with neurosurgical lesions had less than the most favorable outcome (GOS 5), and patients were more likely to have less favorable outcomes thus favoring use of biomarkers.^10^

This difference in GOS health state distribution in these economic analyses along with the assumptions around patients moving between GOS health states post 5 years in the analysis by Pandor and colleagues^23^ resulted in a prediction of lower overall QALYs than we observed. Thus, we believe the derivation of these values and our approach is more conservative and more representative of real-world practice than those derived by Pandor and colleagues and other previous analyses.^10,23,29^

An advantage of our analysis is that we report the impact on CT scans and years lived with favorable outcomes, which are limited (or not reported at all) in other analyses. Although QALYs may be considered a good proxy for favorable outcome, the former outcomes are likely more meaningful to physicians and patients. Although CT scans may not always be available and may increase time to diagnosis, unnecessary use of CT scans exposes patients to radiation, although the impact of radiation exposure on a patient's outcomes has been shown to be minimal. Presenting these outcomes helps physicians understand the potential impact that biomarker tests can have on diagnosis and treatment.

Our analysis is not without limitations. One limitation is that follow-up of patients to understand the long-term clinical and economic impacts (e.g., distribution of patients by GOS scores, annual costs of patients with a GOS status) of an mTBI is limited. Mild TBI is often thought of as a shorter-term injury (1–6 weeks, typically) from which patients seem to make a full recovery. However, mTBI can have long-term effects, such as significant memory and attention problems. Even with a better understanding of these costs and outcomes, our analysis is likely to underestimate the usefulness of a biomarker at the initial step of mTBI evaluation because of the limited data on patient sequelae.

Another limitation of our analysis is that we do not consider incidental findings of CT scans. Specifically, performing a CT could result in finding other diseases or conditions that may require further workup and incur costs. As an example, there were three “false-negative” biomarker panel tests from samples from subjects completing the ALERT-TBI, one of which was later found not to be a TBI injury but rather a vascular defect.^14^ As such, more extensive use of CT scanning could be more costly overall than we have estimated here. Berge and associates denote that “incidental findings are frequent, rarely severe, rarely iatrogenic, and relatively expensive.”^30^ However, even though costly, this can result in a significant benefit for patients. It is important for clinicians to consider these potential benefits when determining their preferred diagnostic workup approach.

In our base-case analysis, the “CT all” scenario assumes that all patients with mTBI get a CT scan upon arrival to the ED along with clinical evaluation as first-line assessment. This is an extreme scenario that may not occur often or at all in reality. According to a recent transnational survey in Southern European countries, French physicians demonstrate a high rate of adherence (70%) to national mTBI management guidelines and criteria for performing CT.^31^ Corresponding CT use in patients with mTBI was perceived by most French physicians to be between 50% and 100%, reflecting that use may be as high as 100%.^31^ We consequently decided to use a 100% CT rate as a base-case comparator in our model because it seems challenging to estimate the real rate of CT use in patients with mTBI, and results may differ between regions, hospitals, and even practitioners within the same hospital.^31^ We acknowledge that our approach may be subject to criticism. Nevertheless, it appears that in other settings most patients with blunt head injury and subsequent symptoms have a head CT scan performed as part of usual care.^21^

We also recognize that our assumption of biomarker screening approach (where all patients with mTBI get a biomarker test) may not be completely realistic. Indeed, only a portion of patients with mTBI may be eligible and benefit from biomarker testing as a part of the CT decision-making process. Scandinavian guidelines recommend performing blood S100B only in low-risk patients with mTBI, whereas high- and medium-risk patients should undergo CT without biomarker testing, and patients with GCS 15 and minimal head trauma could be discharged without CT and without biomarker testing.^7^ Newly released French national guidelines suggest the use of S100B and combination GFAP+UCH-L1 only in patients with mTBI with intermediate risk of ICL, to limit CT use.^32^ Biomarker validation trials, which we used and reference in our model, however, used broader mTBI populations (head CT as part of clinical care), not limited to patients within a particular risk category.^14^ Further studies will be important to evaluate the clinical performances, added value (in comparison with usual care), and cost-effectiveness of using biomarkers in patients with mTBI with particular risks for ICL. We believe that the above approaches are valid in modeling methodology and that their limitations counterbalance each other without favoring biomarker or head CT.

Another limitation is that we did not explicitly consider the impact of clinical decision rules (e.g., Canadian CT Head Rule) along with the use of biomarkers, which have the potential to enhance clinical decision-making.^21^ In actual clinical practice, clinicians would likely use these biomarkers in conjunction with a clinical decision rule or guidelines, which may result in the sensitivity and specificity of using biomarkers alone being underestimated. In a recently published prospective cohort study from a Level 1 U.S. trauma center, the combined use of the Canadian CT Head Rule plus GFAP yielded the highest area under the receiver operating characteristic (AUROC, 0.88 [95% confidence interval, 0.81-0.95]).^21^ Unfortunately, we were unable to calculate the values of sensitivity and specificity of the Canadian CT Head Rule plus GFAP combination from the area under the curve data to use in sensitivity analysis. Such information on combined sensitivity and specificity would be of additional scientific and practical value for the current analysis. Indeed, it is interesting to note that 56% to 61% of emergency physicians from this study noted that they were comfortable with using decision rules and 86% of physicians noted that a biomarker blood test would or might be useful in deciding whether to order a CT scan for a patient.^21^ Understanding this additive effect in conjunction with actual treatment is important, especially because the use of these rules alone has been shown to only have a modest impact on CT use.^33,34^

The most robust algorithm for a clinical decision rule plus biomarker combination (biomarker first then clinical decision rule or clinical decision rule first then biomarker) and the approach for final combination interpretation are subjects of current research.^21^ This research holds great potential in significantly decreasing CT use without compromising patient safety if improved diagnostic performance results especially around specificity. Indeed, in a secondary analysis of 919 patients with a GCS 14–15 who met criteria for Canadian CT Head Rule determination in the ALERT-TBI study, the sensitivity of the Canadian CT Head Rule was 71.2% (lower than that observed in other studies), and the specificity was high at 55.1%.^35^ GFAP+UCH-L1 was 96.2% sensitive and 38.8% specific in the same population of 919 patients.

Further, we assumed CT scans were 100% accurate in diagnosing lesions and determining lesion types. Radiologists may differ in how they interpret CT scans,^30,36,37^ and some lesions may be missed or difficult to see. However, all diagnoses were assumed to consider clinical patient assessment in addition to interpretation of the scan. Incorporating the use of biomarker tests should further help clinicians be more confident in their decision-making.^21^ If assumptions around actual diagnosis were incorrect, the sensitivity analyses confirmed that results were robust to small changes.

In summary, our model showed that with GFAP+UCH-L1 screening, the number of CT scans performed was lower compared with CT scan alone or with S100B. This finding could have a substantial impact on hospitals and treatment protocols, particularly as the incidence and awareness of mTBI grows. Costs, life-years, QALYs, and favorable outcome between the different approaches remain similar. In addition, GFAP+UCH-L1 may be used in a broader mTBI population (larger time window following trauma to perform testing and ability to use also in patients with extracranial injuries) as compared with S100B.

## Supplementary Material

Supplemental data

Supplemental data
