# Supplementary material for: Cost-Effectiveness of Blood-Based Brain Biomarkers for Screening Adults with Mild Traumatic Brain Injury in the French Health Care Setting
Source: J Neurotrauma. 2023 Mar 28;40(7-8):706–19. doi: 10.1089/neu.2022.0270 (PMC10061334; doi:10.1089/neu.2022.0270)
Supplement: Supplemental data [file Suppl_AppendixS1.docx]

Cost-Effectiveness of Blood-Based Brain Biomarkers for Screening Adults With Mild Traumatic Brain Injury in the French Healthcare Setting

Supplementary Materials

# MATERIALS AND METHODS

## Patient Population

The hypothetical cohort of 1,000 patients was limited to those with mild traumatic brain injury (mTBI) with isolated head trauma, as use of the S100B biomarker is limited to this population. This limitation did not apply to patients potentially tested with combination glial fibrillary acidic protein and ubiquitin C-terminal hydrolase-L1 (GFAP+UCH-L1).^1,2^

## Comparators

Standard clinical assessment was assumed to include standard clinical evaluation with Glasgow Coma Scale (GCS) ascertainment of patient medical history and evaluations for conditions such as posttraumatic seizures, focal neurological deficits, and clinical signs of trauma/fracture. Assessment with the GCS^3^ was assumed to be followed by possible use of clinical decision rules and/or guidelines such as those of French Society of Emergency Medicine.^4^ However, it was challenging to estimate the percentage of patients in whom it could have been applied.

## Sensitivity and Specificity

Test sensitivity measured the probability of detecting lesions given that lesions exist, and specificity measured the probability of not detecting lesions given that lesions do not exist. Sensitivity and specificity for the GFAP+UCH-L1 test was examined in a prospective clinical study of adults (≥ 18 years old) presenting to the emergency department (ED) with a suspected mTBI. Sensitivity and specificity in this analysis is for mTBI patients with GCS 13-15 upon which the US Food and Drug Administration’s approval decision was based.^5^ All patients received a computed tomography (CT) scan, and blood was collected for testing within the specified time of injury.

For the alternative set of sensitivity and specificity for GFAP+UCH-L1, specificity for no lesions and sensitivity for nonneurosurgical lesions were estimated using an alternative diagnostic platform for the GCS 13-15 population. Sensitivity for neurosurgical lesion was assumed to be 1.000 (range, 0.800-1.000), as no head CT findings of false-negative subjects were neurosurgically, manageable injuries.^6^

The base and alternative sensitivity for nonneurosurgical lesions and specificity for no lesions of S100B were obtained from their respective sources.^7,8^ The sensitivity for neurosurgical lesions was conservatively assumed to be 1.0.

## Patient Outcomes

To estimate the percentage of patients within each Glasgow Outcomes Scale (GOS) health state, given lesion type and deterioration, we performed a review of clinical studies. These data were extracted similarly to the methods used by Pandor et al.^9^ However, care was taken to ensure outcomes were specific to patients with a GCS score of 13-15.

Patients with no lesions were assumed to have all symptoms resolve such that all patients experienced normal function (i.e., 100% of these patients will have a GOS score of 5).

Patients with nonneurosurgical lesions and no deterioration were assumed to be managed optimally when lesions were detected. As a result, these patients do not deteriorate and are managed such that most patients revert to normal function (GOS = 5). However, a small percentage of patients with nonneurosurgical lesions deteriorate. For these patients, GOS was determined by the number of patients within various GOS health states as seen in Fabbri et al.^10^ and Cheung et al.^11^ Fabbri et al.^10^ examined GOS outcomes in patients with GCS score ≥13. Cheung et al.^11^ examined outcomes in patients with GCS score of 13-15 with nonneurosurgical lesions who did not require immediate surgery. We combined the patients from these two studies to determine the GOS score for these patients.

The percentage of patients within the various GOS health states with neurosurgical lesions who had immediate surgery was estimated from studies by Deverill and Aitken,^12^ Cheung et al.,^11^ and Gerlach et al.^13^ Specifically, patients in Gerlach et al.^13^ had a GCS score of 13-15 and were found to need immediate surgery. These patients were combined with patients from Deverill and Aitken,^12^ who had a GCS score of ≥13 and had access to neurosurgical facilities (i.e., immediate surgery), and patients from Cheung et al.,^11^ who had a GCS score of 13-15 and surgery.

To estimate the percentage of patients within the various GOS health states with neurosurgical lesions with delayed surgery, we adjusted the GOS percentages of patients with neurosurgical lesions with immediate surgery by an increased risk of unfavorable outcome (2.4 times) from Deverill and Aitken,^12^ as seen in patients experiencing a delay in neurosurgery when neurosurgical lesions occurred (i.e., percentage of patients with GOS score <3 was 2.4 times higher). The proportion of patients with GOS scores 4 and 5 was estimated as the remaining patients, with the proportion of patients in GOS 4 and 5 being proportionate to the portion of patients in GOS 4 and 5 in patients with neurosurgical lesions with immediate surgery.

Consistent with an analysis by Pandor et al.,^9^ we allowed patients to move between health states after year 6 in sensitivity analysis. Specifically, Whitnall et al.^14^ followed a cohort of patients admitted to the hospital for a head injury and assessed clinical outcomes at 1 year and 5-7 years. The proportion of patients in various GOS health states at year 1 and again at years 5-7 (assumed to be year 6) was reported.

The percentages reported by Whitnall et al.^14^ were not adjusted for all-cause mortality. Since Whitnall et al.^14^ denoted that the results were not associated with patient characteristics (e.g., age, gender, severity of injury, previous head injury) except mental well-being (i.e., depression, anxiety, stress, and lower self-esteem), we adjusted their outcomes by taking death out of the calculation. The percentage of patients moving from their resultant health state after the index event to GOS 3, 4, or 5 health states was calculated. All patients in GOS 2 were assumed to remain in GOS 2 unless they died because of all-cause mortality.

## Resource Use

Patients who receive biomarker test results denoting lesions or who have comorbidities, signs, or symptoms such that further evaluation or observation is recommended were assumed to receive a CT scan. Specifically, all patients with a test result positive for lesions and with a test result negative for neurosurgical lesions but who further deteriorate received a CT scan. Additionally, 20% of patients with a test negative for lesions and 30% of patients with a test negative for nonneurosurgical lesions were deemed to need 24-hour observation and received a CT scan (clinical opinion).

Patients progressed through the model with the use of various resources. For patients with no lesions regardless of biomarker test outcome, 70% were assumed to be discharged immediately (in patients with positive biomarker results, the absence of the lesions was confirmed by head CT) with no follow-up, whereas the remaining 30% were assumed to remain for observation for < 24 hours. These patients were also assumed to not incur additional issues and thus did not require further follow-up (clinical opinion).

For patients with nonneurosurgical lesions in whom the test did not identify lesions (false-negative result), 50% of patients were assumed to be discharged immediately and 50% were assumed to be held for <24-hour observation. Of those discharged immediately, 10% were assumed to have a follow-up visit with a general practitioner. A further 10% of those visiting the general practitioner were assumed to seek care from a neuropsychologist (clinical opinion). A small fraction of 0.168% of patients experienced deterioration such that they returned to the ED seeking care.^15^ In patients who were kept for observation, we assumed 29.9% had a follow-up visit with a general practitioner. Again, 10% of those visiting the general practitioner sought care from a neuropsychologist (clinical opinion), and 0.112% of patients experienced deterioration such that they returned to the ED.^15^

Patients in whom the tests correctly identified nonneurosurgical lesions (true-positive) were managed more intensively than patients with no lesions found. As a result, we assumed that 70% of these patients would be observed for <24 hours. Twenty percent of these patients were assumed to have a follow-up visit with a general practitioner, and 10% of those visiting the general practitioner sought care from a neuropsychologist (clinical opinion). Ten percent of patients with positive biomarker results were assumed to be admitted to the critical care unit after initial head CT for 1 day, then move to the neurosurgery ward for 2.5 days where they received a CT scan to monitor for stability and an additional CT scan to confirm stability before discharge. These patients had a follow-up CT scan and visit with a neurosurgeon. Twenty percent of patients were assumed to be admitted directly to the neurosurgical ward for monitoring. While in the ward and post discharge, these patients were managed similarly to the patients who were admitted to the critical care unit.

For patients with neurosurgical lesions in whom the test did not identify lesions (false-negative), 15% were assumed to be discharged immediately. The remaining 85% were assumed to require intensive management. Sixty-three percent of those admitted experienced fast clinical deterioration, followed by head CT and immediate surgery then subsequent stay in the critical care unit and neurosurgery ward. Post surgery, these patients incurred an additional CT scan to confirm they were stable. Post discharge, these patients incurred a 1- and 6-month follow-up with a neurosurgeon along with CT scans to ensure these patients were stable.

We assumed that 37% of patients admitted entered the short stay/general ward. However, these patients were assumed to deteriorate prior to discharge and thus incurred a delayed surgery. Post surgery, these patients were managed similarly to patients who had immediate surgery (clinical opinion). The 15% of patients who were discharged immediately were assumed to return to the ED, where they required immediate surgery that should have been performed earlier. They were subsequently managed similarly to the patients who incurred a delayed surgery prior to discharge.

All patients in whom tests properly identified neurosurgical lesions (true-positive) were assumed to incur immediate surgery and postsurgery resources and were managed as other patients who had surgery. Length of stay in the critical care unit was assumed to be 1 day, whereas the length of stay in the neurosurgical ward for patients with neurosurgical lesions was assumed to be 7 days (clinical opinion). The proportions of patients incurring the different resources are presented in Figure 2 of main article.

## Costs

The cost of S100B in France is listed at €32.40.^16^ In the absence of a cost for a test for GFAP+UCH-L1, we assumed parity with S100B.

The CT scan costs include the cost of the scan and radiologist review.^17^ Costs are composed of a technical and medical component where one component can increase overtime while the other component can decrease. For simplicity, CT scan costs were not inflated to 2020 euros. Variability of this cost was examined in sensitivity analysis.

A review of the literature found that French resource use and costs by GOS health state (Table 2 of main article) are limited. As a result, we adapted the resource use and costs for each GOS health state from Pandor et al.^9^ Costs were converted to euros using the Organisation for Economic Co-operation and Development purchasing power parity values between England and France,^18^ then inflated to 2020 euros using Institut National de la Statistique et des Études Économiques.^19^

For patients who experienced radiation-induced cancer, we assumed an annual cost of €10,059, which was obtained from COS Paris Healthcare^20^ and inflated to 2020 euros using Institut National de la Statistique et des Études Économiques.^19^

All costs are presented in 2020 euros.

## Utilities

Utility weights, associated with each GOS health state, were obtained from the published literature and were used to calculate quality-adjusted life-years (QALYs). Utilities measure a person’s perception of well-being under certain health states and range from 0.0 to 1.0, in which a utility of 1.0 represents perfect health and a value of 0.0 represents death.

Glasgow Outcomes Scale health state utilities were obtained from the CT in Head Injury Patients (CHIP) study, which included long-term outcomes from a subset of patients to estimate utility values for GOS health states (Table 2 of main article).^21^ Utilities were derived from EuroQoL-5D. The GOS 2 health state (vegetative state) was not seen in the trial. As a result, we assumed a utility of 0, which is similar to the utility assumed for these patients in Pandor et al.^9^

Each time a patient entered the ED/hospital because of mTBI signs and symptoms, a per visit decrement of 0.012 (95% confidence interval [CI], 0.0050-0.0222) was incurred.^22^ The annual decrement of 0.103 (average of the difference in utilities reported by patients with no condition and all cancer) was incurred when cancer occurred in a patient because of the radiation exposure from a CT scan.^23^

## Model Analysis

Lifetime costs and outcomes were derived. Outcomes included life-years, QALYs, number of CT scans, number of ED visits, and years with favorable outcome (GOS score >3).

One-way sensitivity analyses were performed to examine the impact of each parameter. We examined the effect of changing each parameter in one-way sensitivity analyses, where individual parameters were varied based on 95% CIs, plausible ranges from the literature, or ± 20% when neither CIs nor plausible ranges were available. Results were plotted on tornado diagrams in which inputs were presented from most sensitive to least sensitive.

Scenario analyses include the impact on results when varying lesion prevalence, sensitivity and specificity, utilities, risk of radiation-induced cancer and associated mortality, and change in GOS score post year 5.

Second-order Monte Carlo simulations were run to understand the variability of results. Analyses were run 10,000 times to ensure stability in the results. Means and standard deviations for each comparator were reported. In addition, the mean difference, standard deviation, and 95th percentile interval between GFAP+UCH-L1 and CT scan and between GFAP+UCH-L1 and S100B were reported.

For probabilistic sensitivity analyses, discount rates, percentage of patients using various resources, sensitivity/specificity of the biomarkers, risk of radiation-induced cancer by age, utilities by GOS score, and mortality associated with radiation-induced cancer were varied according to a beta distribution. GOS score distributions and the distribution of the different types of surgeries performed were drawn from Dirichlet distribution. Costs (e.g., diagnostic, health state, surgery, and annual cost of radiation-induced cancers), prevalence of nonneurosurgical lesions, and length of stay in the different wards were drawn from a gamma distribution.

# RESULTS

## Sensitivity Analysis Results

In a scenario in which the prevalence of lesion types varied as reported by Smits et al.,^24^ the differences in number of CT scans and in cost decreased slightly (321.06 versus 325.42 CT scans in the base case and €3,800 versus €4,150 per 1,000 patients in the base case, respectively) when comparing GFAP+UCH-L1 and CT scan. When comparing GFAP+UCH-L1 and S100B, the differences in CT scans and cost also decreased slightly (45.25 versus 46.43 CT scans in the base case and €4,527 versus €4,736 per 1,000 patients in the base case, respectively) versus S100B.

Overall, as the prevalence of neurosurgical lesions increased, the difference in CT scans and costs decreased when using GFAP+UCH-L1 versus CT scan or S100B. The use of GFAP+UCH-L1 always incurred fewer CT scans; however, the costs eventually increased for patients using GFAP+UCH-L1 such that as the prevalence approached 15%, use of CT scanning became less costly.

A scenario analysis in which patients’ GOS score changed post year 5 had little impact on the results. Of course, no change in number of CT scans or ED visits resulted. However, the difference in costs increased when compared with CT scan (€4,223 per 1,000 patients saved versus €4,150 in the base case) and decreased when compared with S100B (€4,692 per 1,000 patients saved versus €4,736 in the base case).

The use of alternative values for utilities resulted in no real difference in results. However, overall QALYs increased when using utilities from Dijkers,^25^ derived by using Health Utilities Index and Kosty et al.^26^ and Tsauo et al.^27^ and derived from interviews of patients with head injuries.

When the risk of radiation-induced cancer and associated mortality is projected based on only the last 2 risk data points, we observe that the incidence and mortality are higher, which favors GFAP+UCH-L1. Thus, we observe that the difference in CT scans remains constant, but the difference in costs increases when compared with CT and S100B. In fact, when radiation-induced cancer is assumed to not occur and impact outcomes, there is no change in the number of CT scans that are performed. The costs for patients receiving either GFAP+UCH-L1 or CT scan decrease, but patients receiving GFAP+UCH-L1 still have lower costs.

Probabilistic results are presented in Table S1.

Table S1. Second-Order Monte Carlo Simulation

| Outcome/model comparator | Base case | Second-order Monte Carlo simulations | | | |
| --- | --- | --- | --- | --- | --- |
|  |  | Mean | Standard deviation | Lower 5th percentile | Upper 95th percentile |
| **Number of scans per 1,000 patients** | | | | | |
| GFAP+UCH-L1 | 770.88 | 774.06 | 81.45 | 772.46 | 775.65 |
| CT scan | 1,096.30 | 1,099.03 | 67.59 | 1,097.70 | 1,100.35 |
| S100B | 817.32 | 820.48 | 79.27 | 818.92 | 822.03 |
| **Difference in number of scans per 1,000 patients** | | | | | |
| GFAP+UCH-L1 versus CT scan | –325.42 | -324.97 | 19.09 | -325.35 | -324.60 |
| GFAP+UCH-L1 versus S100B | –46.43 | -46.42 | 23.29 | -46.88 | -45.96 |
| **Total costs per person** | | | | | |
| GFAP+UCH-L1 | €564.28 | €560.31 | €460.78 | €551.28 | €569.34 |
| CT scan | €568.43 | €564.43 | €461.97 | €555.37 | €573.48 |
| S100B | €569.01 | €565.03 | €460.42 | €556.01 | €574.06 |
| **Difference in total costs per person** | | | | | |
| GFAP+UCH-L1 versus CT scan | -€4.15 | -€4.12 | €26.58 | -€4.64 | €3.59 |
| GFAP+UCH-L1 versus S100B | -€4.74 | -€4.72 | €4.09 | -€4.80 | €4.64 |

CT = computed tomography; GFAP+UCH-L1 = combination glial fibrillary acidic protein and ubiquitin C-terminal hydrolase-L1.

# REFERENCES

1. Undén J, Ingebrigtsen T, Romner B, et al. Scandinavian guidelines for initial management of minimal, mild and moderate head injuries in adults: an evidence and consensus-based update. BMC Med 2013;11(1):50.

2. Bazarian JJ, Biberthaler P, Welch RD, et al. Serum GFAP and UCH-L1 for prediction of absence of intracranial injuries on head CT (ALERT-TBI): a multicentre observational study. Lancet Neurol 2018;17(9):782-789.

3. Vos PE, Alekseenko Y, Battistin L, et al. Mild traumatic brain injury. Eur J Neurol 2012;19(2):191-198.

4. Jehlé E, Honnart D, Grasleguen C, et al. Traumatisme crânien léger (score de Glasgow de 13 à 15): triage, évaluation, examens complémentaires et prise en charge précoce chez le nouveau-né, l’enfant et l’adulte. Annales françaises de médecine d'urgence 2012;2(3):199-214.

5. US Food and Drug Administration. Evaluation of automatic class III designation for Banyan Brain Trauma Indicator decision memorandum. February 14, 2018. <https://www.accessdata.fda.gov/cdrh_docs/reviews/DEN170045.pdf>. [Last accessed: July 26, 2022].

6. Bazarian JJ, Welch RD, Caudle K, et al. Accuracy of a rapid glial fibrillary acidic protein/ubiquitin carboxyl-terminal hydrolase L1 test for the prediction of intracranial injuries on head computed tomography after mild traumatic brain injury. Acad Emerg Med 2021;28(11):1308-1317.

7. Mondello S, Sorinola A, Czeiter E, et al. Blood-based protein biomarkers for the management of traumatic brain injuries in adults presenting to emergency departments with mild brain injury: a living systematic review and meta-analysis. J Neurotrauma 2018.

8. Elecsys® S100. Product characteristics. F. Hoffmann-La Roche Ltd.; October 9, 2019 2019. <https://diagnostics.roche.com/ch/de/products/params/elecsys-s100.html>. [Last accessed: September 10, 2019].

9. Pandor A, Goodacre S, Harnan S, et al. Diagnostic management strategies for adults and children with minor head injury: a systematic review and an economic evaluation. Health Technol Assess 2011;15(27):1-202.

10. Fabbri A, Servadei F, Marchesini G, et al. Observational approach to subjects with mild-to-moderate head injury and initial non-neurosurgical lesions. J Neurol Neurosurg Psychiatry 2008;79(10):1180-1185.

11. Cheung PS, Lam JM, Yeung JH, et al. Outcome of traumatic extradural haematoma in Hong Kong. Injury 2007;38(1):76-80.

12. Deverill J, Aitken LM. Treatment of extradural haemorrhage in Queensland: interhospital transfer, preoperative delay and clinical outcome. Emerg Med Australas 2007;19(4):325-332.

13. Gerlach R, Dittrich S, Schneider W, et al. Traumatic epidural hematomas in children and adolescents: outcome analysis in 39 consecutive unselected cases. Pediatr Emerg Care 2009;25(3):164-169.

14. Whitnall L, McMillan T, Murray G, et al. Disability in young people and adults after head injury: 5–7 year follow up of a prospective cohort study. J Neurol Neurosurg Psychiatry 2006;77(5):640-645.

15. Livingston DH, Lavery RF, Passannante MR, et al. Emergency department discharge of patients with a negative cranial computed tomography scan after minimal head injury. Ann Surg 2000;232(1):126-132.

16. Ministère des Solidarités et de la Santé. Le référentiel des actes innovants hors nomenclature de biologie et d’anatomopathologie (RIHN). 2019. <https://solidarites-sante.gouv.fr/systeme-de-sante-et-medico-social/recherche-et-innovation/rihn>. [Last accessed: January 16, 2020].

17. Securite Sociale l'Assurance Maladie. Landmarks n°50. The activity of liveral doctors through CCAM: Actes techniques médicaux: quantité et montants remboursés correspondants en 2016. 2018. <https://www.ameli.fr/sites/default/files/2018-07_actes-ccam-2016_points-de-repere-50_assurance-maladie.pdf>. [Last accessed: April 7, 2022].

18. Organisation for Economic Co-operation and Development. Purchasing power parities (PPP). 2020. <https://data.oecd.org/conversion/purchasing-power-parities-ppp.htm>. [Last accessed: April 22, 2020].

19. Institut National de la Statistique et des Études Économiques. Harmonised index of consumer prices. 2020. <https://www.insee.fr/en/statistiques/series/103157760?COICOP2016=2319377>. [Last accessed: April 22, 2020].

20. COS Paris Healthcare. Cancer statistics in France: the key facts and figures. 2019. <https://www.cos-eu.com/en/find-cancer-france-cos/cancer-statistics-france/>. [Last accessed: December 11, 2019].

21. Smits M, Dippel DW, Nederkoorn PJ, et al. Minor head injury: CT-based strategies for management--a cost-effectiveness analysis. Radiology 2010;254(2):532-540.

22. Salomon JA, Vos T, Hogan DR, et al. Common values in assessing health outcomes from disease and injury: disability weights measurement study for the Global Burden of Disease Study 2010. Lancet 2012;380(9859):2129-2143.

23. Mittmann N, Trakas K, Risebrough N, et al. Utility scores for chronic conditions in a community-dwelling population. Pharmacoeconomics 1999;15(4):369-376.

24. Smits M, Dippel DW, Steyerberg EW, et al. Predicting intracranial traumatic findings on computed tomography in patients with minor head injury: the CHIP prediction rule. Ann Intern Med 2007;146(6):397-405.

25. Dijkers MP. Quality of life after traumatic brain injury: a review of research approaches and findings. Arch Phys Med Rehabil 2004;85:21-35.

26. Kosty J, Macyszyn L, Lai K, et al. Relating quality of life to Glasgow outcome scale health states. J Neurotrauma 2012;29(7):1322-1327.

27. Tsauo J-Y, Hwang J-S, Chiu W-T, et al. Estimation of expected utility gained from the helmet law in Taiwan by quality-adjusted survival time. Accid Anal Prev 1999;31(3):253-263.
