# Supplementary material for: Cost-Effectiveness of Blood-Based Brain Biomarkers for Screening Adults with Mild Traumatic Brain Injury in the French Health Care Setting
Source: J Neurotrauma. 2023 Mar 28;40(7-8):706–19. doi: 10.1089/neu.2022.0270 (PMC10061334; doi:10.1089/neu.2022.0270)
Supplement: Supplemental data [file Suppl_TableS1.docx]

Table S1. Second-Order Monte Carlo Simulation

| Outcome/model comparator | Base case | Second-order Monte Carlo simulations | | | |
| --- | --- | --- | --- | --- | --- |
|  |  | Mean | Standard deviation | Lower 5th percentile | Upper 95th percentile |
| **Number of scans per 1,000 patients** | | | | | |
| GFAP+UCH-L1 | 770.88 | 774.06 | 81.45 | 772.46 | 775.65 |
| CT scan | 1,096.30 | 1,099.03 | 67.59 | 1,097.70 | 1,100.35 |
| S100B | 817.32 | 820.48 | 79.27 | 818.92 | 822.03 |
| **Difference in number of scans per 1,000 patients** | | | | | |
| GFAP+UCH-L1 versus CT scan | –325.42 | -324.97 | 19.09 | -325.35 | -324.60 |
| GFAP+UCH-L1 versus S100B | –46.43 | -46.42 | 23.29 | -46.88 | -45.96 |
| **Total costs per person** | | | | | |
| GFAP+UCH-L1 | €564.28 | €560.31 | €460.78 | €551.28 | €569.34 |
| CT scan | €568.43 | €564.43 | €461.97 | €555.37 | €573.48 |
| S100B | €569.01 | €565.03 | €460.42 | €556.01 | €574.06 |
| **Difference in total costs per person** | | | | | |
| GFAP+UCH-L1 versus CT scan | -€4.15 | -€4.12 | €26.58 | -€4.64 | €3.59 |
| GFAP+UCH-L1 versus S100B | -€4.74 | -€4.72 | €4.09 | -€4.80 | €4.64 |

CT = computed tomography; GFAP+UCH-L1 = combination glial fibrillary acidic protein and ubiquitin C-terminal hydrolase-L1.
